# The Effect of Exogenous Beta-Hydroxybutyrate Salt Supplementation on Metrics of Safety and Health in Adolescents

**DOI:** 10.3390/nu13030854

**Published:** 2021-03-05

**Authors:** Matthew Stefan, Matthew Sharp, Raad Gheith, Ryan Lowery, Jacob Wilson

**Affiliations:** The Applied Science and Performance Institute, Research Division, Tampa, FL 33607, USA; msharp@theaspi.com (M.S.); rgheith@theaspi.com (R.G.); rlowery@theaspi.com (R.L.); jwilson@theaspi.com (J.W.)

**Keywords:** ketones, Beta-Hydroxybutyrate, ketosis, diet therapy, exogenous ketones, ketogenic diet, safety

## Abstract

The ketogenic diet is a high-fat, very low-carbohydrate, moderate-protein diet that will induce a state of ketosis, but because of its restrictive nature, it may be difficult to adhere to, especially in adolescents. Supplementing with exogenous beta-hydroxybutyrate salts may induce a state of temporary ketosis without any undesirable side effects, thereby promoting the benefits of ketosis and minimizing adherence requirements to a ketogenic diet. To date, beta-hydroxybutyrate supplementation in healthy adolescents has not been explored. Therefore, the purpose of this study was to examine the safety of exogenous beta-hydroxybutyrate salt supplementation in a healthy adolescent population. In the present study, 22 healthy male and female adolescents consumed 3.75 g of beta-hydroxybutyrate salts or maltodextrin placebo twice daily for 90 days. Comprehensive blood safety analysis, bone densitometry, happiness and emotional intelligence surveys, and blood pressure were assessed at Pre, Day 45, and Day 90. There were no significant differences detected in subjects supplementing with beta-hydroxybutyrate salts, indicating that exogenous beta-hydroxybutyrate salts had no detrimental impact on fasting blood safety metrics, bone density, happiness, emotional intelligence, or blood pressure. We conclude that supplementing with exogenous beta-hydroxybutyrate salts is safe and well-tolerated by healthy adolescents.

## 1. Introduction

Previous research has commonly described the ketogenic diet as a diet consisting of 50 g, or less, of carbohydrates per day. This typically equates to 5 to 10 percent of total caloric contribution coming from carbohydrates [1,2]. Protein intake makes up 5 to 20 percent of the diet, while fat intake contributes the largest portion, comprising 75 to 90 percent of the diet [1,2]. The purpose of the ketogenic diet is to mimic a fasted state by inducing ketosis, a metabolic state where blood ketone concentrations are between 0.5 millimolar (mmol/L) and 3.0 mmol/L [3]. Rises in endogenous ketone levels can vary depending on the availability of fuel substrates, glucose and fatty acids, as well as hormonal signaling of glucagon, insulin, and cortisol.

The ketogenic diet has been utilized to reduce the incidence of seizures in adolescents with epilepsy [4]. There is also evidence to support that the ketogenic diet can improve outcomes of other neurological and metabolic-based disorders like attention deficit disorder [5,6], autism [7,8,9,10], control tumor growth of some forms of cancer by limiting glucose availability [11], and lowering glucose and hemoglobin A1c in individuals with type 2 diabetes [12,13]. Mechanistically, the ketogenic diet alters fuel utilization by forcing the body to rely on its internal storage of fatty acids to create endogenous ketones. In turn, this simultaneously alters mitochondrial function, thereby improving metabolic flexibility and countering insulin resistance [14,15]. 

The ketogenic diet is restrictive in nature (carbohydrate restriction) and may have some undesirable side effects which, in turn, can make it difficult to adhere to. Some of the undesirable side effects are gastrointestinal discomfort [16], constipation [4], vomiting [4], lack of energy [4], hunger [4], and increases in apoB-lipoproteins [17]. For that reason, it may be beneficial to have rapid and temporary rises in ketone concentrations with no dietary modifications. As an alternative, or to supplement a well-formulated ketogenic diet, exogenous ketones have been shown to be a reliable method of rapidly increasing blood ketone levels and promoting a state of ketosis [18]. In addition, the safety of exogenous beta-hydroxybutyrate salts (BHB) has only been explored in a healthy adult population and was determined to be safe [19]. Although the ketogenic diet has appeared to be safe and effective in treating epilepsy and reducing the occurrence of seizures in adolescents [4,20], no studies have examined the safety of supplemental ketones (BHB) on a healthy, adolescent population. Therefore, the purpose of this study is to examine the safety of 90 days of exogenous BHB salt supplementation as determined by hematological safety markers, bone densitometry, emotional intelligence and psychological well-being, and cardiovascular markers of health in healthy adolescents.

## 2. Materials and Methods

### 2.1. Subject Criteria

Subjects recruited for the study were healthy male and female adolescents aged 10 to 17 years old who were non-obese subjects (BMI ≤ 30 kg/m^2^), and who demonstrated no underlying health conditions (heart disease, diabetes, kidney disease), no heart defects, asthma, neurological disorders, and did not take medications for attention deficit hyperactivity disorder. This study was approved by an external institutional review board (IntegReview IRB, Austin, TX, USA) and all procedures were in agreement with institutional guidelines and the Declaration of Helsinki. Prior to engagement in any study procedures, written informed content was provided by the parents of the adolescent via assent forms. Descriptive statistics for all subjects are provided below (Table 1).

### 2.2. Study Design

This study was carried out in a randomized, double-blind, placebo-controlled manner. Twenty-four adolescents were randomly divided into 2 groups (50/50 split), BHB and placebo (PLA). There were 6 males, and 6 females randomized into the BHB group. Consequently, there were 5 males and 7 females randomized into the PLA group. Two subjects were lost over the course of the study due to time constraints, 1 male and 1 female, both in the PLA group. Thus, descriptive statistics are provided for the 22 subjects that completed the trial. A serving of 3.75-g of BHB or a similarly flavored placebo, in visually identical packets, was administered twice daily for 90 days. In addition, BHB, the experimental condition, contained: 750 mg of leucine, 112.5 mg of theanine, 375 mg of creatine, and vitamins B6 and B12. Subjects were instructed to consume one serving in the morning prior to any meal, and one serving in the afternoon about an hour and a half after lunch. Subjects were simply asked to consume their specified supplement for the duration of the study (90 days) and report to the laboratory for testing at baseline (Pre), Day 45, and Day 90. Adherence to the supplementation protocol was determined by the amount of packets the subjects returned at subsequent laboratory visits (Day 45 and Day 90). Adherence was determined to be 93.3% for BHB group and 94.4% for the PLA group at the completion of the study. In addition, to ensure that one serving of the exogenous BHB salt induced ketosis (≥0.5 mmol/L), 6 subjects took part in a pilot study (2 males, 4 females; age: 13.3 ± 2.4 years; height: 152.17 ± 11.07 cm; body mass: 46.67 ± 10.23 kg). Lastly, subjects submitted a subjective assessment on emotional intelligence and happiness at Pre, Day 45, and Day 90. Testing procedures are outlined below.

### 2.3. Venous Blood Measures

Venous blood was extracted by venipuncture of the antecubital vein using a 21-gauge syringe and collected into a 10 mL EDTA vacutainer tube (BD Vacutainer^®^, Becton, Dickinson and Company, Franklin Lakes, NJ, USA) by a certified phlebotomist. Afterward, blood samples were centrifuged at 2500 rpm for 10 min at 4 °C. Resulting plasma samples were then aliquoted and stored at −80 °C until further analysis. Samples were thawed once and analyzed in duplicate in the same assay for each analysis to avoid compounded inter-assay variance. 

### 2.4. Bone Densitometry and Body-Composition Analysis

Body mass was measured to the nearest 0.1 kg using a digital scale, and the subject’s height was verbally confirmed (Seca, Chino, CA, USA). Total and regional body composition was determined by a whole-body scan on a dual-energy X-ray absorptiometry device (DXA) (Horizon A DXA System; Hologic Inc, Marlborough, MA, USA). Each subject was scanned by a certified technician, and the digital segmentation was determined via computer algorithm. Fat-free mass, fat mass, and body fat percentage was determined for each scan. The subject was asked to wear comfortable clothing and remove any items that could attenuate the X-ray beams (this could be jewelry, items containing wire, shoes, etc.). The subject was asked to lie in a supine position with knees and elbows extended and instructed not to move for the entire duration of the scan (approximately 5 min). The DXA has a switching-pulse system that rapidly alternates the voltage of the X-ray generator, producing two beams of high and low energies. The attenuated X-rays that have passed through the subject are measured sequentially with a detector situated on the scanning arm above the patient. An internal wheel corrects for any small fluctuations caused by this method of beam generation. Results from each scan were uploaded and accessed on a computer that was directly linked to the DXA device. Calibration of the densitometer on the DXA device was done against a phantom provided by the manufacturing company prior to testing. The abdominal region was delineated by an upper horizontal border located at half of the distance between the acromion processes and external end of iliac crests, a lower border determined by the external end of iliac crests and the lateral borders extending to the edge of the abdominal soft tissue. 

### 2.5. Blood Pressure and Heart Rate

Subjects rested in a supine position for 5 min in a quiet room at 22.8 °C before the baseline hemodynamic measurements were obtained. Resting brachial blood pressure and heart rate were measured on the right arm with an automated digital oscillometric sphygmomanometer (Omron, Model HEM 705-CP; Omron Corporation, Shimogyo-ku, Kyoto, Japan). Three readings separated by 1-min intervals were taken, and the mean was used for the analysis. 

### 2.6. Happiness and Emotional Intelligence Surveys

The Trait Meta Mood Scale (TMMS-24) has been used to evaluate perceived emotional intelligence. The questionnaire is formed by 24 items with a Likert-type five-point answer scale (1 = Do not agree, 5 = Totally agree). Three dimensions are evaluated (eight items per dimension): attention (ability to feel and express feelings appropriately); clarity (understanding of emotional states); and repair (appropriate emotional regulation). Each dimension can be classified into three traits depending on the score: Attention; (1) Attention should be improved, (2) Adequate attention, (3) Excessive attention; Clarity; (1) Clarity should be improved, (2) Adequate clarity, (3) Excellent clarity; Repair; (1) Repair should be improved, (2) Adequate repair, (3) Excellent repair.

The objective of the Oxford Happiness Questionnaire is to measure happiness in general (i.e., psychological well-being). A series of statements about happiness are given and the participants indicate their degree of agreement with each one. In psychometric terms, it consists of 29 items or 29 potential sources of happiness and the participants consider the extent to which they form part of their experiences. It employs a six-point Likert-type scale (1 = I totally disagree, 6 = I totally agree). The lowest score that can be obtained is 1 (if Answer 1, ‘I totally disagree’ is chosen in all the statements) and the highest is 6 (if Answer 6; ‘I totally agree’ is chosen for all the statements) [21,22]. 

### 2.7. Statistical Analysis

All statistical analyses were performed at the completion of the study using GraphPad Prism (Version 9, San Diego, CA, USA). Dependent variables were assessed for normality (Shapiro–Wilk test) and homogeneity of variances (Levene’s test). Two-way mixed model analysis of variance (ANOVA) was performed assuming group as the between-subject factor, time as the within-subject factor, and subjects as a random factor. Whenever a significant F value was obtained, a post hoc test with a Bonferroni adjustment was used to for multiple comparisons purposes. The alpha level was set at *p* ≤ 0.05. Data are reported as mean ± standard deviation. Additionally, we reported the mean difference, upper and lower limit values of 95% confidence intervals (95% CI) of the mean difference for between-group comparisons. Lastly, between-group effect sizes (*d*) were calculated as [(Mean2 − Mean1)/SD_Pooled_).

## 3. Results

### 3.1. Blood Ketone Pilot Data

**Figure 1:**
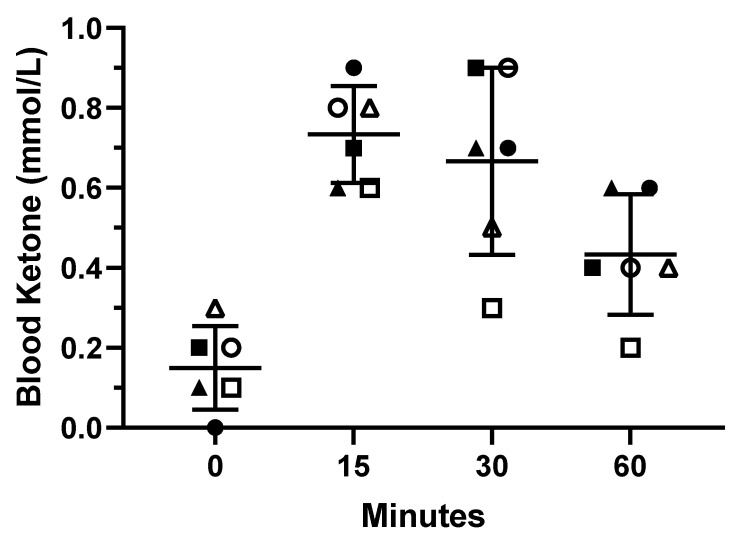
Individual Blood Ketone. Response. Horizontal bars represent mean and standard deviation values. Each unique data point style represents a single participant.

At baseline, zero out of six subjects were in ketosis (0.2 ± 0.1 mmol/L). At 15 min postprandial exogenous BHB salt consumption, six out of six (100%; blood ketones: 0.7 ± 0.1 mmol/L) subjects had reached a state of ketosis. At 30 min postprandial exogenous BHB salt consumption, five out of six (83%; blood ketones: 0.7 ± 0.2 mmol/L) subjects had maintained a state of ketosis. At 60 min post prandial exogenous BHB salt consumption, two out of six (33%; blood ketones: 0.4 ± 0.2 mmol/L) subjects had maintained a state of ketosis. Individual responses are displayed in Figure 1.

### 3.2. Complete Blood Count

There was no significant between- or within-group differences in the Complete Blood Count values (*p* ≥ 0.05, Table 2). Mean and standard deviation are displayed in Table 2.

### 3.3. Automated Differential Cell Count

There was no significant between- or within-group differences in any values of Automated Differential Cell Count (*p* ≥ 0.05, Table 3). Mean and standard deviation are displayed in Table 3.

### 3.4. Comprehensive Metabolic Panel

A significant group by time interaction was detected for Albumin:Globulin Ratio, Creatinine, and Carbon Dioxide (*p* ≤ 0.05, Table 4). A post hoc analysis was carried out for Albumin:Globulin Ratio, Creatinine, and Carbon Dioxide and it indicated that there were no significant differences between groups (*p* ≥ 0.05). However, in the PLA, Albumin: Globulin Ratio was significantly higher at Day 45 compared to Pre (Mean Diff: 0.27 U/L; 95% CI: 0.03, 0.52; *p* = 0.03, *d* = 0.73) and Day 90 (Mean Diff: 0.36 U/L; 95% CI: 0.12, 0.61; *p* = 0.002, *d* = 0.91) (*p* < 0.05). No differences were observed in BHB (*p* ≥ 0.05). For Creatinine, values at Day 45 were significantly lower than Pre (Mean Diff: −0.10 mg/dL; 95% CI: −0.15, −0.05; *p* = 0.0001, *d* = 1.09) and Day 90 (Mean Diff: −0.10 mg/dL; 95% CI: −0.15, −0.06; *p* = 0.0001, *d* = 1.25) for the PLA group whereas no significant changes were detected in BHB (*p* ≥ 0.05). For Carbon Dioxide, values at Day 90 were significantly greater than Day 45 (Mean Diff: 1.70 mL/min; 95% CI: 0.23, 3.18; *p* = 0.02, *d* = 0.90) in the PLA group and no changes were found in BHB (*p* ≥ 0.05). Despite these statistically significant differences in Albumin:Globulin Ratio, Creatinine, and Carbon Dioxide, no values exceeded their respective reference ranges indicating no clinical significance. There was no significant between- or within-group differences for the remaining values of the Comprehensive Metabolic Panel (*p* ≥ 0.05, Table 4). Mean and standard deviation are displayed in Table 4. 

### 3.5. Body Composition

There were no significant between- or within-group differences in any Body Composition or Bone Densitometry measure (*p* ≥0.05, Table 5). 

### 3.6. Blood Pressure and Heart Rate

There was no significant between- or within-group differences in resting blood pressure or heart rate (*p* ≥ 0.05, Table 6). Mean and standard deviation are displayed in Table 6.

### 3.7. Happiness and Emotional Intelligence Surveys

There was no significant between- or within-group differences for responses to the Qxford Health Questionnaire or the Trait Meta Mood Scale (TMMS-24) (*p* ≥ 0.05, Table 7). Mean and standard deviation are displayed in Table 7.

## 4. Discussion

In the present study, we demonstrated the safety of exogenous BHB salts under conditions of everyday life in healthy adolescents. The primary finding of this study was that sustained 7.5 g of daily exogenous BHB salt consumption for 90 days was safe for healthy adolescents and had no adverse effect on hematological safety markers, bone densitometry, emotional intelligence and psychological well-being, or cardiovascular markers of health. The findings of this study support and further build upon previous research done in healthy adults [18,19], demonstrating that 90 days of exogenous BHB salt supplementation was considered safe. Secondly, a pilot study was performed to ensure that one serving (3.75 g of exogenous BHB salt) induced a metabolic state of ketosis (≥0.5 mmol/L). Our results indicated that a single 3.75 g serving was able to rapidly increase circulating ketones by 15 min postprandial, and temporarily maintain a state of ketosis for 30 min after consuming exogenous BHB salts. Some individuals may even be able to maintain ketosis for up to 60 min. Therefore, we can conclude that a single serving (3.75 g) induced mild ketosis.

The ketogenic diet, ketosis, and exogenous ketones elicit some controversial views. Some of these views are important to adolescents as they are still growing and developing. One of those views is that ketosis can increase the risk of complications in the kidneys [23] and potentially stunt growth via loss of calcium and electrolytes [23]. Protein content on a ketogenic diet is formulated to be 20% of total caloric intake, however, the ketogenic diet may be formulated to be considered a high-protein diet (consuming more than 0.8 g/kg/day of protein). Previous research has suggested that diets high in protein, like the ketogenic diet, could lower pH and increase the acidic load on the kidneys [23]. Due to the fact that both a ketogenic diet and a high protein diet can be acidic independently, it is plausible that either diet could increase the acidic load on the kidneys. However, Kossof et al. [24] demonstrated that a typically formulated ketogenic diet did not increase the risk of kidney stones in adolescents. The present study corroborated Kossof et al. [24] as there were no changes in kidney function markers such as blood urea nitrogen (BUN), and BUN:Creatinine Ratio, or alteration in acid-base balance. Creatinine and carbon dioxide levels remained unaltered in the BHB condition while these levels varied slightly in the PLA throughout the study. Given that the change in PLA was still within reference ranges, these changes could have occurred from normal physiological variations impacted by sleep or lifestyle habits, like nutritional intake and/or physical activity. In addition, it is well documented in the literature that high-sugar, high-glycemic boluses (e.g., the maltodextrin placebo) have negative impacts on parameters of health in adolescents [25,26,27]. This could further explain the variations in Albumin:Globulin Ratio, Creatinine, and Carbon Dioxide demonstrated at Day 45 in the PLA group only, and not in the BHB group.

Another consideration is calcium loss. This is especially important in adolescents as they are still developing and growing bone structure. Previous research has demonstrated that the ketogenic diet can increase calcium excretion, which could lead to bone-mass loss in adolescents [28,29,30]. The present study demonstrated no blood calcium or electrolyte loss as all electrolyte levels were unchanged over 90 days in healthy adolescents. Moreover, we found no changes in bone mineral density. This was the first study examining the effects of BHB salts on bone mineral density in adolescents. The results suggest that BHB salt supplementation did not alter bone development in the adolescent population.

The present study did not find that the exogenous ketone salt supplementation had any statistically significant impact on blood glucose or hemoglobin A1c. However, there was a trend that both glucose and hemoglobin A1c were declining in the BHB group, while PLA was either staying constant, or increasing slightly, although neither was outside of clinical ranges. The results of our study contradicts that of previous research that found that ketone supplementation can reduce blood glucose levels and improve vascular function in obese adults [31]. The participants in the present study were young, healthy adolescents free of any metabolic dysfunction or irregularities, while the previous study mentioned used obese adults who are more likely to have irregular metabolism, dysfunction, and insulin resistance. These aforementioned pre-existing conditions could influence the efficacy of exogenous ketones on these metabolic irregularities. In a healthy population, these conditions do not exist, minimizing the impact that exogenous ketones may have on blood glucose and hemoglobin A1c.

Lastly, electrolytes play an important regulatory role in cardiovascular health markers such as blood pressure and heart rate [32]. The exogenous BHB used in the present study is bound to sodium, calcium, and magnesium salts. This is done to improve transport and absorption across the gut-blood barrier, and, therefore, improving the efficiency and efficacy of the ketone. Consequently, we found it reasonable to investigate if the consumption of the exogenous BHB bound to these electrolytes would have an effect on blood pressure. Previous research has found that IV infusion of ketone salts raised heart rate [33,34]. However, the present study found that systolic blood pressure, diastolic blood pressure, heart rate, and oxygen saturation were unaffected in the resting state. This contrary finding to previous research may be attributed to the ingestion method, health, and age of the subjects, and the combination of ketone salts with glucose that was infused. Additionally, the previous research examined acute effects, while the current study examined chronic effects of supplementation. Lastly, the current study found that all electrolytes monitored (sodium, potassium, chloride, and calcium) remained unaltered.

A limitation of the present study is the distribution method of supplementation, followed by the lack of dietary and activity control. Subjects were provided with a 45-day supply of the supplement at every laboratory visit. To keep subjects accountable and strengthen adherence, subjects were asked to return any unused supplements. In addition, the supplement was provided to the parents of the adolescents so that they would be responsible for supplementation adherence. 

## 5. Conclusions

To date, there is no data examining the safety of exogenous BHB salt supplementation in healthy adolescents. The purpose of this study was to examine the safety of 90 days of BHB salt supplementation in healthy adolescents. The results indicate that exogenous BHB salt supplementation can induce mild ketosis with no negative effects on any investigated metrics of safety and well-being. Therefore, the conclusion of this study suggests that exogenous BHB salt supplementation can be considered safe for healthy adolescents. Future research may seek to examine the impact of exogenous BHB salts on physical and cognitive performance in adolescents, as well as examine the safety and well-being effects of exogenous BHB salts in an elderly population.

## Figures and Tables

**Table 1:** Descriptive Subject Characteristics.

	BHB (*n* = 12)	PLA (*n* = 10)
**Males/Females (n)**	6/6	4/6
**Age (years)**	13.00 ± 2.61	14.00 ± 2.05
**Height (cm)**	154.31 ± 21.14	160.91 ± 10.05
**Body Mass (kg)**	52.67 ± 20.78	56.35 ± 11.96
**Body Mass Index (kg/m^2^)**	21.46 ± 5.36	21.61 ± 3.45

**Table 2:** Complete Blood Count.

	Pre	*d* (95% CI)	Day 45	*d* (95% CI)	Day 90	*d* (95% CI)	*p*-Value
**WBC (K/uL)**							
**BHB**	6.62 ± 1.74	0.62(−2.81, 0.60)	6.33 ± 1.14	0.62(−2.75, 0.66)	6.48 ± 1.22	0.04 (−1.65, 1.76)	0.0999
**PLA**	7.72 ± 1.81		7.38 ± 2.11		6.43 ± 1.61		
**RBC (M/uL)**							
**BHB**	4.75 ± 0.23	0.06(−0.30, 0.33)	4.79 ± 0.28	0.25(−0.24, 0.40)	4.71 ± 0.19	0.39(−0.42, 0.21)	0.0620
**PLA**	4.73 ± 0.43		4.71 ± 0.35		4.81 ± 0.31		
**Hemoglobin (g/dL)**							
**BHB**	14.12 ± 0.49	1.04(0.03, 1.54)	14.20 ± 0.49	1.32(0.24, 1.75)	13.98 ± 0.55	0.88(−0.16, 1.35)	0.1194
**PLA**	13.33 ± 0.96		13.21 ± 0.94		13.38 ± 0.79		
**Hematocrit (%)**							
**BHB**	41.58 ± 1.23	0.41(−1.21, 2.98)	41.88 ± 1.69	0.66(−0.74, 3.45)	40.63 ± 1.91	0.45(−1.27, 2.92)	0.7144
**PLA**	40.70 ± 2.77		40.52 ± 2.37		39.8 ± 1.81		
**MCV (fl)**							
**BHB**	87.83 ± 2.66	0.32(−3.58, 6.64)	87.50 ± 3.92	0.18(−4.21, 6.01)	86.33 ± 4.09	0.64(−1.88, 8.34)	0.0830
**PLA**	86.30 ± 6.11		86.60 ± 6.08		83.10 ± 5.86		
**MCH (pg)**							
**BHB**	29.80 ± 1.72	0.62(−0.87, 3.77)	29.71 ± 1.36	0.65(−0.81, 3.83)	29.71 ± 1.30	0.94(−0.52, 4.12)	0.6868
**PLA**	28.35 ± 3.11		28.20 ± 3.00		27.91 ± 2.39		
**MCHC (g/dL)**							
**BHB**	33.98 ± 1.19	0.86(0.05, 2.34)	33.93 ± 0.54	1.30(0.19, 2.48)	34.41 ± 0.75	0.94(−0.36, 1.94)	0.5386
**PLA**	32.78 ± 1.58		32.60 ± 1.34		33.62 ± 0.92		
**RDW (%)**							
**BHB**	12.15 ± 0.55	0.97(−1.89, 0.19)	12.20 ± 0.45	0.78(−1.79, 0.29)	12.12 ± 0.49	0.65(−1.83, 0.26)	0.9048
**PLA**	13.00 ± 1.11		12.95 ± 1.29		12.90 ± 1.64		
**Platelets (k/uL)**							
**BHB**	305.58 ± 53.63	0.10(−68.45, 54.62)	282.42 ± 42.79	0.40(−81.02, 42.05)	293.83 ± 51.58	0.17(−70.80, 52.27)	0.5519
**PLA**	312.50 ± 81.45		302.9 ± 59.23		303.10 ± 59.59		

Data reported in mean and standard deviation. *p*-value is from group by time interaction effect. WBC: White blood Cell, RBC: Red Blood Cell, MCV: Mean Cell Volume, MCH: Mean Cell Hemoglobin, MCHC: Mean Cell Hemoglobin Concentration, RDW: Red Cell Distribution Width. 95% CI = 95% confidence interval of the mean difference between groups. *d* = Cohen’s d between-group effect size [(BHB-PLA)/SD_pooled_].

**Table 3:** Automated Differential Cell Count.

	Pre	*d* (95% CI)	Day 45	*d* (95% CI)	Day 90	*d* (95% CI)	*p*-Value
**Neutrophils (%)**							
**BHB**	47.33 ± 7.02	1.04(−16.11, −0.03)	53.17 ± 8.01	0.28(−10.47, 5.61)	47.08 ± 6.56	0.82(−13.36, 2.72)	0.3080
**PLA**	55.40 ± 8.41		55.60 ± 9.18		52.40 ± 6.42		
**Lymphocytes (%)**							
**BHB**	40.67 ± 7.83	0.77(−1.70, 13.83)	33.92 ± 8.13	0.06(−7.35, 8.18)	40.83 ± 7.46	0.83(−2.63, 12.90)	0.2219
**PLA**	34.60 ± 8.03		33.50 ± 7.19		35.70 ± 4.59		
**Monocytes (%)**							
**BHB**	8.42 ± 1.44	1.13(−0.08, 2.92)	9.42 ± 1.68	1.30(0.62, 3.62)	8.67 ± 1.37	0.43(−0.93, 2.07)	0.0583
**PLA**	7.00 ± 1.05		7.30 ± 1.57		8.10 ± 1.29		
**Eosionophil (%)**							
**BHB**	2.67 ± 1.07	0.19(−1.77, 2.30)	2.58 ± 1.68	0.17(−2.45, 1.62)	2.42 ± 1.44	0.25(−2.52, 1.55)	0.2503
**PLA**	2.40 ± 1.71		3.00 ± 3.06		2.90 ± 2.29		
**Basophil (%)**							
**BHB**	0.92 ± 0.29	0.98(0.02, 0.81)	0.92 ± 0.29	0.98(0.02, 0.81)	1.00 ± 0.00	0.67(−0.20, 0.60)	0.3695
**PLA**	0.50 ± 0.53		0.50 ± 0.53		0.80 ± 0.42		
**Granulocytes (%)**							
**BHB**	0.00 ± 0.00	0.44(0.00, 0.00)	0.00 ± 0.00	0.44(0.00, 0.00)	0.00 ± 0.00	0.44(0.00, 0.00)	-
**PLA**	0.10 ± 0.32		0.10 ± 0.32		0.10 ± 0.32		
**Neutrophils (k/uL)**							
**BHB**	3.18 ± 1.21	0.88(−2.54, 0.17)	3.43 ± 1.10	0.51(−2.15, 0.56)	3.03 ± 0.64	0.40(−1.73, 0.98)	0.2945
**PLA**	4.36 ± 1.47		4.22 ± 1.92		3.41 ± 1.17		
**Lymphocytes (k/uL)**							
**BHB**	2.65 ± 0.74	0.06(−0.57, 0.65)	2.11 ± 0.32	0.79(−0.89, 0.33)	2.68 ± 0.77	0.64(−0.21, 1.00)	0.0644
**PLA**	2.61 ± 0.60		2.39 ± 0.39		2.28 ± 0.43		
**Monocytes (k/uL)**							
**BHB**	0.54 ± 0.14	0.00(−0.15, 0.15)	0.61 ± 0.15	0.57(−0.07, 0.23)	0.58 ± 0.14	0.48(−0.09, 0.22)	0.3383
**PLA**	0.54 ± 0.16		0.53 ± 0.13		0.51 ± 0.15		
**Eosionophil (k/uL)**							
**BHB**	0.18 ± 0.09	0.07(−0.15, 0.14)	0.15 ± 0.09	0.49(−0.21, 0.07)	0.18 ± 0.14	0.00(−0.14, 0.15)	0.0716
**PLA**	0.19 ± 0.17		0.22 ± 0.18		0.18 ± 0.13		
**Basophil (k/uL)**							
**BHB**	0.05 ± 0.05	0.2(−0.04, 0.06)	0.03 ± 0.05	0.00(−0.05, 0.06)	0.06 ± 0.05	0.2(−0.05, 0.06)	0.9618
**PLA**	0.04 ± 0.05		0.03 ± 0.05		0.05 ± 0.05		
**Granulocytes (k/uL)**							
**BHB**	0.00 ± 0.00	0.00(−0.01, 0.01)	0.00 ± 0.00	0.47(−0.02, 0.01)	0.00 ± 0.00	0.00(−0.01, 0.01)	0.3047
**PLA**	0.00 ± 0.00		0.01 ± 0.03		0.00 ± 0.00		

Data reported in mean and standard deviation. *p*-value is from group by time interaction effect. 95% CI = 95% confidence interval of the mean difference between groups. *d* = Cohen’s d between-group effect size [(BHB-PLA)/SD_pooled_].

**Table 4:** Comprehensive Metabolic Panel.

	Pre	*d* (95% CI)	Day 45	*d* (95% CI)	Day 90	*d* (95% CI)	*p*-Value
**Total Protein (g/dL)**							
**BHB**	7.15 ± 0.36	0.59(−0.57, 0.17)	7.14 ± 0.40	0.00(−0.38, 0.37)	7.12 ± 0.36	0.06(−0.36, 0.39)	0.2638
**PLA**	7.35 ± 0.32		7.14 ± 0.36		7.10 ± 0.37		
**Albumin (g/dL)**							
**BHB**	4.77 ± 0.24	0.30(−0.36, 0.21)	4.73 ± 0.30	0.64(−0.43, 0.14)	4.68 ± 0.27	0.28(−0.20, 0.37)	0.1068
**PLA**	4.84 ± 0.22		4.90 ± 0.23		4.60 ± 0.31		
**Globulin (g/dL)**							
**BHB**	2.38 ± 0.29	0.39(−0.47, 0.21)	2.40 ± 0.27	0.48(−0.20, 0.48)	2.43 ± 0.31	0.21(−0.41, 0.27)	0.0935
**PLA**	2.51 ± 0.37		2.24 ± 0.39		2.50 ± 0.36		
**ALB: GLOB (U/L)**							
**BHB**	2.03 ± 0.31	0.19(−0.30, 0.41)	2.01 ± 0.26	0.66(−0.59, 0.12)	1.96 ± 0.29	0.25(−0.28, 0.43)	0.0444 **
**PLA**	1.97 ± 0.31		2.27 ± 0.49		1.88 ± 0.36		
**Bilirubin (mg/dL)**							
**BHB**	0.43 ± 0.18	0.37(−0.13, 0.26)	0.50 ± 0.19	0.50(−0.08, 0.32)	0.41 ± 0.11	0.26(−0.15, 0.25)	0.6286
**PLA**	0.36 ± 0.20		0.40 ± 0.21		0.36 ± 0.25		
**Alkaline Phosphate (U/L)**							
**BHB**	242.58 ± 91.52	0.84(−21.27, 173.40)	241.00 ± 86.64	0.67(−34.16, 160.6)	250.67 ± 97.95	0.99(−4.49, 190.2)	0.1283
**PLA**	166.50 ± 90.43		176.78 ± 104.35		157.80 ± 89.02		
**AST (U/L)**							
**BHB**	20.67 ± 6.26	0.32(−15.15, 4.69)	19.83 ± 4.84	0.36(−8.39, 11.45)	19.17 ± 5.95	0.29(−8.45, 11.39)	0.2306
**PLA**	25.90 ± 22.01		18.11 ± 4.73		17.70 ± 4.08		
**ALT (U/L)**							
**BHB**	14.58 ± 3.23	0.01(−5.92, 6.09)	13.25 ± 1.82	1.30(−3.16, 8.86)	12.83 ± 2.48	1.32(−2.37, 9.64)	0.4695
**PLA**	14.50 ± 14.05		10.44 ± 2.46		9.20 ± 3.01		
**BUN (mg/dL)**							
**BHB**	14.17 ± 3.49	1.06(−0.43, 6.97)	13.92 ± 4.27	0.85(−0.48, 6.92)	13.92 ± 4.27	1.04(0.42, 7.82)	0.7229
**PLA**	10.90 ± 2.64		11.00 ± 2.35		9.8 ± 3.61		
**Creatinine (mg/dL)**							
**BHB**	0.75 ± 0.13	0.00(−0.14, 0.14)	0.73 ± 0.17	0.70(−0.06, 0.22)	0.75 ± 0.16	0.00(−0.14, 0.14)	0.0037 **
**PLA**	0.75 ± 0.11		0.63 ± 0.11		0.75 ± 0.08		
**BUN:Creatinine (mg/dL)**							
**BHB**	19.58 ± 6.17	0.92(−2.39, 11.96)	20.25 ± 8.88	0.28(−4.32,10.02)	19.67 ± 8.49	0.89(−0.81, 13.54)	0.1816
**PLA**	14.80 ± 4.06		18.22 ± 5.36		13.30 ± 5.48		
**Sodium (mg/dL)**							
**BHB**	140.17 ± 1.27	0.08(−1.94, 1.47)	139.83 ± 1.85	0.10(−1.57, 1.84)	139.75 ± 1.96	0.51(−0.65, 2.75)	0.2786
**PLA**	140.40 ± 1.01		139.67 ± 1.22		138.70 ± 2.17		
**Potassium (mg/dL)**							
**BHB**	3.90 ± 0.20	0.88(−0.47, 0.09)	4.11 ± 0.19	0.50(−0.39, 0.17)	4.18 ± 0.28	0.71(−0.55, 0.01)	0.4095
**PLA**	4.09 ± 0.23		4.20 ± 0.17		4.45 ± 0.46		
**Chloride (mg/dL)**							
**BHB**	102.08 ± 1.83	0.65(−2.06, 2.43)	100.83 ± 2.44	0.23(−2.91, 1.56)	101.33 ± 2.87	0.03(−2.31, 2.18)	0.7150
**PLA**	103.00 ± 0.83		101.33 ± 1.80		101.40 ± 1.87		
**Carbon Dioxide (mL/min)**							
**BHB**	21.67 ± 1.44	0.98(−0.39, 2.72)	20.33 ± 0.89	0.09(−1.42, 1.69)	20.75 ± 1.36	0.62(−2.71, 0.41)	0.0218 **
**PLA**	20.50 ± 0.88		20.22 ± 1.39		21.90 ± 2.24		
**Calcium (mg/dL)**							
**BHB**	9.56 ± 0.22	0.00(−0.35, 0.35)	9.63 ± 0.36	0.47(−0.44, 0.25)	9.91 ± 0.36	0.15(−0.41, 0.29)	0.8553
**PLA**	9.56 ± 0.12		9.79 ± 0.32		9.97 ± 0.46		
**Glucose (mg/dL)**							
**BHB**	91.83 ± 5.79	0.36(−4.68, 10.94)	87.67 ± 5.73	0.35(−9.74, 5.88)	90.67 ± 8.05	0.17(−6.64, 8.98)	0.3855
**PLA**	88.70 ± 10.99		90.22 ± 8.72		89.50 ± 5.17		
**Hemoglobin A1c (%)**							
**BHB**	5.08 ± 0.17	1.06(−0.36, 0.01)	4.99 ± 0.14	1.61(−0.43, −0.08)	4.99 ± 0.16	1.52(−0.42, −0.07)	0.3375
**PLA**	5.26 ± 0.17		5.25 ± 0.18		5.24 ± 0.17		

Data reported in mean and standard deviation. *p*-value is from group by time interaction effect. ALB: GLOB = Albumin:Globulin Ratio, AST = aspartate aminotransferase, ALT = alanine transaminase, BUN = blood urea nitrogen. 95% CI = 95% confidence interval of the mean difference between groups. d = Cohen’s d between-group effect size [(BHB-PLA)/SD_pooled_].

**Table 5:** Body Composition & Bone Densitometry Results.

	Pre	*d* (95% CI)	Day 90	*d* (95% CI)	*p*-Value
**Total Mass (kg)**					
**BHB**	52.67 ± 20.78	0.22(−21.09, 13.74)	53.47 ± 21.28	0.20(−20.91, 13.93)	0.8056
**PLA**	56.35 ± 11.96		56.96 ± 11.48		
**Fat Mass (kg)**					
**BHB**	15.40 ± 7.87	0.04(−6.69, 7.31)	15.12 ± 7.78	0.03(−6.79, 7.20)	0.6726
**PLA**	15.09 ± 5.96		14.92 ± 5.80		
**Fat Free Mass (kg)**					
**BHB**	37.27 ± 13.98	0.35(−16.04, 8.07)	38.32 ± 14.62	0.31(−15.78, 8.33)	0.6797
**PLA**	41.26 ± 8.51		42.04 ± 8.78		
**Body Fat (%)**					
**BHB**	28.29 ± 6.08	0.31(−4.59, 8.62)	27.38 ± 6.20	0.23(−5.07, 8.14)	0.1220
**PLA**	26.28 ± 7.03		25.85 ± 7.29		
**Bone Mineral Density (g/cm^2^)**					
**BHB**	0.91 ± 0.13	0.62(−0.21, 0.05)	0.92 ± 0.13	0.72(−0.22, 0.04)	0.0590
**PLA**	0.99 ± 0.13		1.01 ± 0.12		

Data reported in mean and standard deviation. *p*-value is from group by time interaction effect. 95% CI = 95% confidence interval of the mean difference between groups. d = Cohen’s d between-group effect size [(BHB-PLA)/SD_pooled_].

**Table 6:** Blood Pressure and Cardiovascular Results.

	Pre	*d* (95% CI)	Day 45	*d* (95% CI)	Day 90	*d* (95% CI)	*p*-Value
**Systolic BP (mmHg)**							
**BHB**	112.33 ± 12.18	0.01(−12.57, 12.84)	109.43 ± 14.43	0.26(−15.67, 9.74)	107.83 ± 13.32	0.23(−15.37, 10.04)	0.6188
**PLA**	112.20 ± 9.58		112.80 ± 11.49		110.50 ± 9.56		
**Diastolic BP (mmHg)**							
**BHB**	61.42 ± 9.18	0.04(−9.79, 10.43)	57.92 ± 9.12	0.06(−10.69, 9.53)	58.67 ± 9.26	0.26(−7.34, 12.88)	0.5225
**PLA**	61.10 ± 8.85		58.50 ± 9.16		55.90 ± 11.88		
**Heart Rate (bpm)**							
**BHB**	72.17 ± 8.43	0.42(−5.54, 13.48)	73.17 ± 7.73	1.30(1.06, 20.08)	70.92 ± 9.52	0.55(−4.29, 14.73)	0.1067
**PLA**	68.20 ± 10.37		62.60 ± 8.47		65.70 ± 9.60		
**Oxygen Saturation (%)**							
**BHB**	98.67 ± 0.65	0.56(−0.75, 1.49)	98.67 ± 0.65	0.59(−0.35, 1.89)	98.75 ± 0.62	0.54(−0.47, 1.77)	0.5485
**PLA**	98.30 ± 0.67		97.90 ± 1.73		98.10 ± 1.60		

Data reported in mean and standard deviation. *p*-value is from group by time interaction effect. BP = Blood Pressure. 95% CI = 95% confidence interval of the mean difference between groups. *d* = Cohen’s d between-group effect size [(BHB-PLA)/SDpooled].

**Table 7:** Happiness and Emotional Intlligence Survey Results.

	Pre	*d* (95% CI)	Day 45	*d* (95% CI)	Day 90	*d* (95% CI)	*p*-Value
**Oxford Questionnaire**							
**BHB**	3.75 ± 0.29	0.19(−0.32, 0.48)	3.72 ± 0.16	0.30(−0.48, 0.33)	3.69 ± 0.34	0.10(−0.37, 0.44)	0.5657
**PLA**	3.67 ± 0.52		3.79 ± 0.29		3.65 ± 0.43		
**TMMS−24 Attention**							
**BHB**	3.22 ± 0.52	0.23(−0.88, 0.57)	3.14 ± 0.75	0.13(−0.63, 0.83)	3.00 ± 0.73	0.31(−0.94, 0.51)	0.5704
**PLA**	3.38 ± 0.82		3.04 ± 0.76		3.22 ± 0.70		
**TMMS−24 Clarity**							
**BHB**	3.61 ± 0.59	0.03(−0.80, 0.83)	3.41 ± 0.67	0.05(−0.86, 0.77)	3.31 ± 0.82	0.35(−1.15, 0.48)	0.3640
**PLA**	3.59 ± 0.63		3.45 ± 0.97		3.64 ± 1.04		
**TMMS−24 Repair**							
**BHB**	3.34 ± 0.52	0.05(−0.66, 0.70)	3.21 ± 0.54	0.11(−0.75, 0.61)	3.38 ± 0.67	0.16(−0.57, 0.79)	0.6800
**PLA**	3.31 ± 0.66		3.28 ± 0.75		3.27 ± 0.74		

Data reported in mean and standard deviation. *p*-value is from group by time interaction effect. TMMS:24: Trait Meta Mood Scale 24. 95% CI = 95% confidence interval of the mean difference between groups. *d* = Cohen’s d between-group effect size [(BHB-PLA)/SD_pooled_].
